# Impact of pet dog or cat exposure during childhood on mental illness during adolescence: a cohort study

**DOI:** 10.1186/s12887-022-03636-0

**Published:** 2022-10-05

**Authors:** Anne Gadomski, Melissa B. Scribani, Nancy Tallman, Nicole Krupa, Paul Jenkins, Lawrence S. Wissow

**Affiliations:** 1 Research Institute , Bassett Medical Center , Cooperstown, NY USA; 2 Data Analyst, Center for Biostatistics, Bassett Research Institute, Cooperstown, NY USA; 3 Data Manager, Center for Biostatistics, Bassett Research Institute, Cooperstown, NY USA; 4 Center for Biostatistics, Bassett Research Institute, Cooperstown, NY USA; 5 Vice Chair for Child and Adolescent Psychiatry, Division Chief, Child Psychiatry and Behavioral Medicine, Department of Psychiatry, University of Washington, Seattle, WA USA; 6 Bassett Research Institute, Bassett Medical Center , Cooperstown, United States

**Keywords:** Pet exposure in childhood, Mental illness in adolescence, Pet attachment, Pet dog, Pet cat

## Abstract

**Background:**

In our prior study of 643 children, ages 4–11 years, children with pet dogs had lower anxiety scores than children without pet dogs. This follow-up study examines whether exposure to pet dogs or cats during childhood reduces the risk of adolescent mental health (MH) disorders.

**Methods:**

Using a retrospective cohort study design, we merged our prior study database with electronic medical record (EMR) data to create an analytic database. Common MH diagnoses (anxiety, depression, ADHD) occurring from the time of prior study enrollment to 10/27/21 were identified using ICD-9 and ICD-10 codes. We used proportional hazards regression to compare time to MH diagnoses, between youths with and without pets. From 4/1/20 to 10/27/21, parents and youth in the prior study were interviewed about the amount of time the youth was exposed to a pet and how attached s/he was to the pet. Exposure included having a pet dog at baseline, cumulative exposure to a pet dog or cat during follow-up, and level of pet attachment. The main outcomes were anxiety diagnosis, any MH diagnosis, and MH diagnosis associated with a psychotropic prescription.

**Results:**

EMR review identified 571 youths with mean age of 14 years (range 11–19), 53% were male, 58% had a pet dog at baseline. During follow-up (mean of 7.8 years), 191 children received a MH diagnosis: 99 were diagnosed with anxiety (52%), 61 with ADHD (32%), 21 with depression (11%), 10 with combined MH diagnoses (5%). After adjusting for significant confounders, having a pet dog at baseline was associated with lower risk of any MH diagnosis (HR = 0.74, p = .04) but not for anxiety or MH diagnosis with a psychotropic prescription. Among the 241 (42%) youths contacted for follow-up, parent-reported cumulative exposure to pet dogs was borderline negatively associated with occurrence of any MH diagnosis (HR = 0.74, p = .06). Cumulative exposure to the most attached pet (dog or cat) was negatively associated with anxiety diagnosis (HR = 0.57, p = .006) and any MH diagnosis (HR = 0.64, p = .013).

**Conclusion:**

Cumulative exposure to a highly attached pet dog or cat is associated with reduced risk of adolescent MH disorders.

## Background

Nearly 1 in 3 US teens are diagnosed with an anxiety disorder [1–4]. Anxiety disorders in adolescence and adulthood often begin in childhood with a median age of onset at 11 years [1–3]. Anxiety is also often comorbid with, or even misclassified as, Attention Deficit Hyperactivity Disorder (ADHD) in children and teens, so current estimates may under-estimate its true prevalence [5]. Anxious children often develop impaired social relationships, academic underachievement, substance abuse, and depression [6–8]. While early treatment of childhood anxiety is effective, only about half the children with any mental health (MH) disorders are treated due to limited access to MH therapy [9–11]. The COVID-19 pandemic has exacerbated these pre-existing MH challenges, thus increasing the prevalence of elevated anxiety and depression symptoms among children and adolescents [12, 13].

Preventing or mitigating evolution of MH problems in childhood to MH disorders in adolescence and later life would be very impactful, yet little is known on what prevention strategy may work [14]. Pet dogs may ameliorate childhood anxiety through emotional support and attachment [15–19]. Dogs have long been recognized as salient agents for child emotional development because they follow human communicative cues [15, 16]. The psychological support that pets provide may be greatest during childhood and early adolescence [18]. Youth challenged by psychosocial, developmental and self-esteem transitions may be supported by pets before and during adolescence [20, 21].

Our prior cross-sectional study of 643 children recruited in a primary care clinic showed that children, ages 4 to 11 years, with pet dogs had lower anxiety scores (for separation and social anxiety) than children without pet dogs [22]. That study used tablet screening tools at pediatric annual visits to determine how pet dogs may affect child mental and physical health. The mean scores for the Screen for Child Anxiety Related Emotional Disorders (SCARED-5) that is based on Diagnostic and Statistical Manual, Fourth Edition (DSM-IV) symptoms were 1.13 (1.00, 1.26) for children with pet dogs and 1.40 (1.23, 1.58) for children without (p = .01). The proportion of children with a score ≥ 3, for which more in-depth diagnostic work is indicated, was 12% for children with pet dogs compared to 21% of children without pet dogs (p = .002). We also found that, in a secondary analysis focused on pet cat ownership controlling for child age, poverty, and parental depression, pet cat ownership was associated with *more* attention problems (b = 1.38, SE = 0.53, p < .01) [23].

Divergent results such as these in studies of human animal interaction (HAI) are often attributed to small sample sizes, lack of adjustment for confounding factors, misinterpretation of correlational factors as causal factors, and inability to randomize subjects to experimental or control conditions [24–27]. Prospective studies that control for a wide range of confounders are needed [28], but have also led to inconsistent results as illustrated by the following examples. A longitudinal birth cohort study of 627 children in the US showed that prenatal exposure to pet dogs, but not cats, was associated with twice the odds of ADHD in 10 to 12 year old boys [29]. A longitudinal study of 3017 children in Australia found that pet dog or cat ownership was associated with a small positive effect on the socioemotional development of young children [30]. As with our prior study [23], children with cats had higher hyperactivity scores compared to children not owning any pets (OR, 1.40; 95% CI, 1.08–1.80). A cohort study in Japan showed that dog exposure (but not cat exposure) at six months of life was associated with decreased risk of developmental delay among infants 12 months of age [31].

The strength of a child’s attachment to the pet, rather than pet ownership per se, may confer benefit. While the underlying mechanisms are unclear, attachment theory stipulates a bidirectional connectivity between humans and their pets that may exert a significant influence on a child’s socioemotional development [32–34]. The degree of attachment between a child and a pet dog or cat is associated with improved child social-cognitive and socioemotional development, and psychosocial health [18, 35, 36]. However, longitudinal studies of the impact of children’s attachment to pet dogs or cats on youth MH are limited.

Building upon our prior studies as well as addressing limitations of HAI studies and the potential role of child pet attachment, we investigated what impact child/youth HAI may have on adolescent MH. We sought evidence of whether exposure to pet dogs or cats during childhood is inversely related to the occurrence of adolescent MH disorders. Specifically, using the electronic medical record (EMR) to determine the time to first MH disorder diagnosis, the aim of this study was to compare the incidence density of MH diagnosis between youths with pet dog and/or cat exposure to those without pet exposure. Additional aims were to measure the association between pet exposure and the occurrence of sub-threshold or threshold but undiagnosed MH disorders over the follow-up period, and to explore how attachment and potential confounders affect the relationship between pet dog and/or cat exposure and youth MH.

## Methods

In a retrospective cohort study design, clinical data for this study was extracted from Bassett Healthcare Network’s system-wide integrated Epic EMR (Epic Systems Inc., Verona, WI) that has been used in this health network since 2011. Our prior study database was merged with EMR data to create a patient-level analytic database that included common adolescent MH diagnoses (anxiety, depression, ADHD) occurring from the time of enrollment in the prior study to 10/27/21. In addition, parents and youth included in the original study were contacted to collect data on subsequent pet exposure and pet attachment data, covariates (demographics etc.), and youth MH scales to measure anxiety, depression, and social support. In order to account for the differential follow-up time among the subjects, time to event and incidence density were the main outcomes of the study rather than cumulative incidence.

### EMR data abstraction for MH Diagnoses

MH diagnoses of children and adolescents are increasingly considered categorical siloes that do not easily lend themselves to developmental trajectory, maturation, progression, extension or resolution [37]. Nevertheless, MH diagnoses do signify a functional problem, a clinical need and/or troublesome symptoms in a child or adolescent’s life that are brought to medical attention. MH diagnoses of interest in this study population included anxiety, any common MH diagnoses and MH diagnosis associated with psychotropic medication, assuming that acquiring an MH diagnosis in the EMR was a significant event reflecting functional limitation or troublesome symptoms. International Classification of Disease, Ninth Edition, Clinical Modification (ICD-9) codes before 10/1/15, and ICD-10 codes thereafter, were used to identify the first occurring MH diagnoses among all EMR encounters. Diagnosis and prescription orders are structured data fields in the Epic EMR. These data were drawn from all outpatient and inpatient encounters (including pediatric, general medical, emergency department, surgical, dental and specialty mental health). Previous studies have shown that EMR prescription data for children has very high quality whereas ICD-9/ICD-10 codes may be more variable but still sensitive [38, 39]. In addition, validation studies of EMR ICD-9 code of 314.X for ADHD have shown that these codes are highly sensitive in identifying ADHD cases [40].

Psychotropic medication prescribing was defined as evidence of any oral psychotropic medication prescription included in Epic orders. These included antidepressant, anxiolytic/sedative-hypnotic, antipsychotic, and medications for ADHD (including stimulants, atomoxetine, selective alpha-2 receptor agonists), but excluded anticonvulsants used for seizure treatment, amitriptyline used for migraine prophylaxis and pre-procedure benzodiazepine. Anticonvulsants (carbamazepine, lamotrigine, valproate/divalproex) were included as psychotropic medications if the patient did not have a seizure disorder diagnosis. Prescriptions for psychotropic medication were retrieved not only to validate the MH diagnosis but also to create the category of MH diagnosis prescribed a psychotropic medication, as a proxy for more severe MH disorders for youth seen in primary care.

After MH diagnosis and medication extraction, 10% of EMR records with an MH diagnosis and all records with psychotropic prescription were validated by manual full text EMR encounter review to resolve any discrepancies in date of diagnosis or prescription, or reason for prescription use. These cases were reviewed with a consulting child psychiatrist (LSW) to determine whether they met inclusion criteria. Children who had an MH diagnosis at baseline, i.e. before or on the date of enrollment in the original study 8 years ago, were excluded from the proportional hazards regression analysis. Post hoc exclusion criteria of MH diagnoses made during the follow-up period included: (i) chronic, serious MH diagnoses unlikely to be significantly impacted by presence of a pet dog or cat, (ii) operative dental encounters with the diagnosis of dental anxiety, and no subsequent visits with anxiety diagnosis, and (iii) anxiety visit diagnosis associated with physical exams, procedures, ED visits and no subsequent visits with an anxiety diagnosis.

### Follow-up interview and survey data collection

This part of the study gathered self-reported data from parents and youth ~ eight years after the prior study. Re-recruitment was planned to occur before, during or after primary care clinic visits as had occurred in the prior study. Unfortunately, the onset of the COVID-19 pandemic coincided with the start of this study so in-person recruitment was not possible. Instead, research staff contacted prior study participants by phone and/or email in the same order as they were recruited previously to arrange a time for telephone interview. After informed consent was obtained from the parent and youth, the parent was interviewed about the youth’s pet exposure over the past 8 years. Then the parent and youth completed a secure, HIPAA compliant, digital questionnaire. Data from these questionnaires were downloaded on the secure Research Electronic Data Capture (REDCap) [41] server at the Bassett Research Institute. Covariates included demographics (education, employment status, income level), number of children at home, the index child’s birth order, asthma/allergy status and adverse experiences with dogs or cats, and family history of mental illness or autism. The following rating scales were included:

*Screen for Child Anxiety Related Emotional Disorders* (SCARED41) includes dimensional measures of anxiety symptoms and is used to assess general anxiety, separation anxiety, social phobia, school phobia and physical symptoms of anxiety. It has 41 items that measure these dimensions with cut-off values for each [42, 43].

*Adverse Child Experiences* (ACE-Q) questionnaire yields a cumulative score of stressors (as opposed to revealing specific stressors). The 10 item ACE–Q score was completed by the parent who indicated the number of ACE related statements that pertained to the index youth [44]. Aggregate-level versus item-level reporting was utilized because parents may be more likely to disclose ACEs when this level of privacy is provided [45].

*Patient Health Questionnaire 8-item* (PHQ-8) is a commonly used screener for depression, a condition that is frequently co-morbid with anxiety [46, 47].

*Companion Animal Bonding Scale* (CABS) has 8 items that measure attachment to a companion animal using behavioral interactions between people and their pets [48, 49]. This questionnaire was completed for all pet dogs and cats by the parent, whereas the youth only completed the CABS for the pet with the highest parent rated CABS.

*Multidimensional Scale of Perceived Social Support* (MSPSS) is designed to measure perceptions of support from 3 sources: Family, Friends, and a Significant Other [50]. It has 12 items, with 4 items for each of the three subscales. Mean MSPSS scores 1-2.9 are considered low support; 3–5 moderate support and 5.1-7 high support.

Study participants were mailed gift cards after completion of the interview and questionnaires.

### Statistical analysis

Demographics and other characteristics were compared across study groups using chi-square for sex, Fisher’s Exact test for race, and the t-test for age. Categorical data collected via questionnaire, such as residence, rating scales, etc. were compared across study groups using chi-square test or Fisher’s Exact test as necessary. These tests were used to assess whether known or potential sociodemographic confounders were associated with both pet exposure and time to MH diagnosis. All covariates were tested to see if they were statistically associated with both MH diagnosis and pet exposure; if associated with both, they were included in all final proportional hazards regression models. Missing values for the household income variable were imputed using a multiple imputation Markov chain Monte Carlo method. This method was employed because the missing value pattern was not monotonic. Spearman’s rank correlation coefficient was used to measure the correlation between categorical or ordinal variables.

#### EMR derived MH diagnosis

We used proportional hazards regression to analyze (1) time to anxiety diagnosis, (2) time to any common MH diagnosis and (3) time to any MH diagnosis associated with a psychotropic prescription. Models two and three excluded subjects with any MH diagnosis preceding baseline and included 571 youth. Model one excluded only subjects diagnosed with anxiety prior to baseline and included 605 youth. Pet exposure, defined as the presence of the pet dog at baseline, was derived from the baseline study and was coded as yes or no. Proportional hazard models were adjusted for age, sex, zip code poverty level and baseline SCARED5 (score ≥ 3 on the 5 item SCARED5 completed by the parent for the child at the time of the original study). Results were reported as adjusted hazards ratios (HR) with 95% confidence intervals (CI).

#### EMR plus 8 year follow-up data

Models for youth who completed 8 year follow-up were additionally adjusted for potentially confounding covariates. Confounders were selected based on having a significant association with dog or cat ownership (exposure) and time to MH diagnosis (outcome). The mean and median follow-up time was calculated as the time between enrollment in our prior study and the follow-up phone call ~ eight years later.

#### Pet exposure

Based on parental interview at the ~ 8 year follow-up, cumulative pet exposure variables were constructed for dogs and/or cats. The cumulative value includes the number of days of pet dog and/or cat exposure and the number of these pets during follow-up as a fixed value established for each subject. Cumulative exposure was designed as a measure of the child’s exposure to dogs over the entire course of follow-up expressed in units of 100%. For example, if one dog was present with the child over the entire course of follow-up, the cumulative exposure would be 100% (for 2 dogs the entire time = 200% etc.) If a child was splitting his/her time evenly between two households and a dog was present at one household for the entire follow-up, this child would be assigned a value of 50%. Changes in exposure that occurred over the course of follow-up were also accounted for. For example, if a dog in the child’s home was lost for some reason 75% of the way through follow-up, the child would be assigned a value of 75%. Similarly, if two dogs were present and one was lost half way through follow-up with the other being present the entire time, the child was assigned a value of 150%. Similar logic was applied if dogs were added to the child’s household partway through follow-up.

#### Pet attachment

At follow-up, the parent CABS and youth CABS for the most attached pet were highly correlated (n = 226, Spearman’s rho = 0.80, p < .0001). Regardless of the amount of pet exposure time, the pet with the highest CABS score per the parent was classified as the child’s most attached pet. Children without pets were excluded from this model. The hazard ratio was expressed as a function of a 50% increase in the presence of the most attached pet (the range for this pet exposure was 0 to 100% with a mean of 54%). An interaction term of the type of pet (cat versus dog) by cumulative exposure was tested and was not statistically significant in the prediction of time to MH diagnosis, and was therefore dropped. The final proportional hazards model included a main effect for type of pet in addition to the cumulative exposure.

All statistical analyses were performed using SAS version 9.4 statistical software (Cary, NC).

## Results

EMR analysis revealed 643 subjects who had been in the prior study. Fourteen children did not have a subsequent visit, therefore these subjects had their follow-up time set to zero, leaving 629 subjects available for follow-up analysis.

### Exclusions

Given that a MH diagnosis was found prior to the follow-up period, 56 children with any pre-existing MH diagnoses were excluded from further analysis. These diagnoses were ADHD (42/56, 75%), anxiety (10/56, 18%), anxiety and depression (2/56, 3.6%), other serious emotional disorders (2/56, 3.6%). Four additional subjects with chronic, serious MH diagnosis unlikely to be significantly mitigated by the presence of a pet dog or cat were excluded from analysis. There were 12 subjects missing SCARED5 score at baseline who were excluded, leaving 571 in the proportional hazards regression analysis (Table 1).

**Table 1:** Baseline Covariates for 571 youth included in modeling of pet dog at baseline

Covariate	Total	With pet dog(s) at baseline	Without pet dog(s) at baseline	P value (with pet dog vs. without)
**Youth characteristics**				
# (%) ^a^	571	330 (57.79)	241 (42.21)	
Child age, years Mean (SD)	6.57 (2.13)	6.60 (2.13)	6.53 (2.14)	0.6913
# (% ) male	304 (53.24)	175 (53.03)	129 (53.53)	0.9065
SCARED ≥ 3 (%) at baseline ^b^	73 (12.78)	33 (10.00)	40 (16.60)	0.0197
**PARENT or Guardian Characteristics**				
# (%) relationship: mother^c^	451 (80.25)	250 (76.92)	201 (84.81)	0.0204
Mean % (SD) subjects in baseline zip code living in poverty	14.76 (5.40)	14.94 (5.36)	14.50 (5.47)	0.3370
# (%) parent/guardians with positive PHQ at baseline^d^	10 (1.81)	4 (1.25)	6 (2.56)	0.3360

^a^ Total sample size is 571, that excludes 60 subjects with pre-existing/complicated MH diagnoses and excludes 12 subjects with missing baseline SCARED5.

^b^ This statistical difference was published in a prior study (see Reference 22)

^c^ Nine parent/guardians did not report on relationship to child

^d^ 19 parent/guardians did not have baseline PHQ score

### Inclusions

Six subjects with anxiety diagnosis associated with physical exams, procedures, ED visits, dental extraction, and no subsequent visits with anxiety diagnosis were included, but these encounters with anxiety diagnoses were excluded. There were 34 instances of psychotropic medications that were not associated with any visit MH diagnosis; these encounters were included as any MH diagnoses associated with a psychotropic medication.

Among the 571 youth included in the EMR analysis, 97.8% reside in a county that is a Mental Health Professional Shortage Area (MHPSA). Overall, 55.8% of non-dog owning households at baseline became dog owners during the follow-up period.

### Covariate analysis

Table 2 compares covariates between those who completed follow-up by phone (241) and those who did not. Importantly, the incidence density (per 100 child-years) of MH diagnosis among those who completed follow-up was 5.89 and not significantly different from 4.98 to 100 child-years among those without follow-up (p = .27). Youth who had a pet dog at baseline were more likely to complete follow-up 8 years later (p = .03). Otherwise, the groups were similar.

**Table 2:** Comparison of 571 youth who did or did not complete eight year follow-up (fup) interview based on baseline covariates

Covariate	Completed 8 year fup interview	Did not complete fup interview	P value (fup vs. no fup)
#	216^a^	355	
Mean (SD) youth age in years	14.1 (2.3)	14.0 (2.1)	0.7995
% male	53.2	53.2	0.9999
% pet dog at baseline	64.4	54.4	0.0335
SCARED ≥ 3 (%) at baseline	10.2	14.4	0.1468
% mothers responded at baseline	84.0	77.9	0.0780
Mean % population in current zip code living in poverty	14.4	15.0	0.2433
% parent PHQ positivity	1.4	2.0	0.7495
Incidence density of MH diagnosis (per 100 child-years)	5.89	4.96	0.2627
# (%) Anxiety diagnosis	44 (51.2)	55 (52.4)	0.9058
# (%) ADHD diagnosis	28 (32.5)	33 (31.4)	
# (%) Depression diagnosis	10 (11.6)	11 (10.5)	
# (%) Combined MH diagnosis	4 (4.7)	6 (5.7)	

^a^216 completed 8 year follow-up does not match the 241 who completed phone follow-up due to exclusion of prior MH diagnoses and 2 subjects with missing baseline SCARED5.

Table 3 shows the covariate associations with cumulative dog exposure for parents and youth who completed follow-up interview 8 years after the baseline study. The only true confounders related to both exposure and outcome were reported income (with imputed values) and type of health insurance. Birth order and history of child having asthma/allergies were associated with cumulative dog exposure (p < .05), but were NOT associated with time to any MH diagnosis. The total MSPSS and subscale scores were not associated with pet exposure. MSPSS was not associated with time to MH diagnosis (HR = 0.987, p = .0945), however a higher level of social support from friends subscale was significantly associated with decreased risk of MH diagnosis (HR = 0.821, p = .0082). Parent-reported household income, imputed for missing values, was associated with cumulative dog exposure and time to MH diagnosis and thus was included in all final models. Public insurance was associated with lower cumulative dog exposure (p = .0159) and was also associated with MH diagnosis (HR = 2.02, p = .0021). Public insurance was highly correlated with income (having public insurance was associated with a higher proportion of subjects in lower income categories; p < .0001 by chi-square), therefore only income was included as a confounder in the final models.

**Table 3:** Covariate associations with cumulative dog exposure for parents and youth who completed follow-up interview 8 years after the baseline study

Covariate	Total	Spearman Correlation Coefficient for Cumulative Dog Exposure	t-statistic (*F-value)	P value
**YOUTH**				
#	241^b^			
Mean (SD) youth age in years	14.82 (2.29)	0.077	--	0.2550
% male	55.51		0.69	0.4903
Mean (SD) of children in the home	2.23 (1.63)	-0.041	--	0.5302
Mean (SD) Birth order of index child	1.69 (1.08)	0.147	--	0.0313
% full time live with parent	94.17		2.02	0.0616
% with public insurance	38.01		-2.43	0.0159
% teens who have asthma or allergies	27.66		2.29	0.0238
% teens who ever had a bad experience with dog or cat	11.49		0.53	0.6025
% Pet dog at T1 ^a^	61.22		8.92	< 0.0001
Mean (SD) SCARED41 score at follow-up	19.50 (16.19)	0.042	--	0.5206
Mean (SD) PHQ8 score at follow-up	4.00 (5.16)	-0.021	--	0.7491
Mean (SD) MSPSS score at follow-up	70.00 (13.62)	-0.056	--	0.3875
Mean (SD) ACE-Q score at follow-up	1.11 (1.41)	-0.023	--	0.7288
**PARENT or GUARDIAN**				
Mean (SD) parent age in years	43.40 (6.74)	0.003	--	0.9680
% mothers	91.29		1.40	0.1761
% married and living together	70.95		1.53	0.1278
% who had a dog or cat growing up (Always/Usually)	80.08		1.26*	0.2857
% working full time^c^	66.39		0.48*	0.6204
% some college education or higher	80.91		-1.56	0.1235
% rating their own health as excellent or very good	67.22		0.22	0.8285
% Hispanic or Latino	1.70		-1.69	0.1847
% white	96.27		0.09	0.9329
% Annual household income > FPL for 4-person household ^d^	94.19		2.27*	0.0489
% live in a house	87.97		-0.73	0.4721
% live in a city or near urban area	1.24		-1.57	0.2503
% with family history of mental illness	29.77		1.08	0.2810
% with autism in family member	13.16		1.19	0.2432
Mean (SD) PHQ8 score at follow-up	3.00 (4.46)	0.079	--	0.2211
**Summary Statistics**	**Mean (SD)**	**Median (IQR)**	**Range**	
Cumulative Dog Exposure	91.65 (78.96)	87.49 (15.38-149.85)	0-316.28	
Cumulative Cat Exposure	74.57 (102.23)	27.31 (0-119.16)	0-684.37	

^**a**^excludes all outdoor housed dog and cats. ^**b**^ 241 is the total number of subjects who completed phone follow up. 19 were excluded from MH models due to MH diagnosis at baseline and 2 excluded due to missing baseline SCARED5. ^c^ Asked during COVID-19 pandemic so may not be representative of pre-pandemic conditions. ^d^ The lowest income category used was the cutoff for federal poverty level (FPL) for a 4 person household (0–26,200).The F-statistic and p-value relate to the association of dog exposure with the full range of income levels (not just dichotomous +/- FPL).

*F-values presented for variables with more than two levels

### Pet dog at baseline

Proportional hazards regression modeling of time to any MH diagnosis based on the presence of a pet dog at baseline included 571 adolescents (mean age 14 years, range 11 to 19 at follow-up), 53% were male, 58% had a pet dog at baseline. During follow-up (mean of 7.8 years), 191 children received a MH diagnosis. Of these, 99 were diagnosed with anxiety (52%), 61 with ADHD (32%), 21 with depression (11%) and 10 with combined MH diagnoses (5%). These models were adjusted for relevant covariates i.e. age, sex, baseline SCARED 5 ≥ 3 and baseline percent of population living in poverty within the zip code [51] (this variable served as a proxy for household income that was not assessed at baseline) (Table 4). The sample sizes included in these models vary based on the diagnostic outcome being modeled and whether the baseline SCARED5 was missing. Having a pet dog at baseline was associated with reduced risk for the occurrence of any MH diagnosis (HR = 0.74, p = .04, 95% CI 0.56–0.99), but not for anxiety diagnosis (HR = 0.88, p = .44, 95% CI 0.63–1.22) or any MH diagnosis associated with a psychotropic prescription (HR = 0.79, p = .23, 95% CI 0.55–1.15). Presence of a pet cat was not specifically ascertained at baseline.

**Table 4:** Adjusted proportional hazards regression modeling of risk of mental health diagnosis over the eight-year follow-up period associated with pet exposure

	Pet Dog at baseline^a^			Cumulative exposure to pet dog^b^			Cumulative Exposure to pet cat^b^			Cumulative exposure to most attached pet^b,c^		
	**N**	**HR (95% CI)**	**p**	**N**	**HR (95% CI)**	**p**	**N**	**HR (95% CI)**	**p**	**N**	**HR (95% CI)**	**p**
Anxiety diagnosis	605	0.88 (0.63–1.22)	0.44	233	0.84 (0.60–1.17)	0.30	233	0.93 (0.71–1.21)	0.58	220	0.57 (0.38–0.85)	0.006
Any MH diagnosis	571	0.74 (0.56–0.99)	0.04	220	0.74 (0.54–1.02)	0.06	220	0.87 (0.68–1.11)	0.28	204	0.64 (0.44–0.91)	0.013
Any MH diagnosis with psychotropic prescription	571	0.79 (0.55–1.15)	0.23	220	0.83 (0.57–1.21)	0.33	220	0.78 (0.56–1.10)	0.16	204	0.69 (0.44–1.08)	0.106

^a^adjusted for age, sex, zip code level poverty and baseline SCARED 5 ≥ 3

^b^adjusted for baseline SCARED5 ≥ 3 and household income

^c^limited to subjects with at least one pet during follow-up

### Cumulative exposure to pet dog or cat during follow-up

Of those 241 youth who completed phone follow-up, 19 were excluded from MH models due to the presence of an MH diagnosis at baseline and two more were excluded due to a missing baseline SCARED5. In this group, there were 75 MH diagnosis associated with visits made during the follow-up period. The distribution of diagnoses was similar to the larger group of 571 subjects, in that, 52% were diagnosed with anxiety (n = 39), 35% with ADHD (n = 26), 9% with depression (n = 7) and 4% with combined MH diagnoses (n = 3). Proportional hazards regression of time to any MH diagnosis based on the cumulative exposure to a pet dog during the eight year follow-up included 220 teens (mean age 14.8 years, range 11 to 19), 53.2% were male, 62.7% had a pet dog at baseline. The mean length of follow-up time was 7.8 and the median was 7.7. Only 21 subjects had no pet exposure during the 8-year follow-up period. Using cumulative exposure to pet dogs reported by the parent during follow-up and adjusting for baseline SCARED5 and self-reported income, pet dog(s) were marginally associated with a reduced risk of any MH diagnosis (HR = 0.74, p = .06, 95% CI 0.54–1.02), but pet cats were not (HR = 0.87, p = .28, 95% CI 0.68–1.11) (Table 4). Neither pet dogs (HR = 0.84, p = .30, 95% CI 0.60–1.17) nor cats (HR = 0.93, p = .58, 95% CI 0.71–1.21) were associated with a reduced risk of an anxiety diagnosis or any MH diagnosis associated with a psychotropic medication.

### Most attached pet

Cumulative exposure to the most highly attached pet dog or cat exposure was associated with a reduced risk for the occurrence of anxiety diagnosis (HR = 0.53, p = .002, 95% CI 0.36–0.79) and any MH diagnosis (HR = 0.58, p = .0032, 95% CI 0.41–0.83), but not for any MH diagnosis associated with a psychotropic medication (Table 4). After adjustment for income, a significant protective effect of the most attached pet for anxiety (HR = 0.57, p = .006, 95% CI 0.38–0.85) and any MH diagnosis (HR = 0.64, p = .013, 95% CI 0.44–0.91) remained. These HR’s indicate that exposure to most attached pet during follow-up is associated with a 43% reduced risk of anxiety and 36% reduced risk in any MH diagnosis. While pet type (dog vs. cat) was not a statistically significant covariate in this model for most attached pet, the variable was retained as a means to adjust for type of pet.

### Screening results for youth without a MH diagnosis

Because youth in this study resided in MHPSA’s and thus could be at a higher risk of not being diagnosed with MH disorder, we examined the association between pet exposure and the occurrence of sub-threshold or threshold but undiagnosed MH disorders over the follow-up period. For 241 youth with self-reported SCARED41 and PHQ8 at the time of follow-up, there was no correlation between cumulative dog exposure and these total scores (for SCARED41 Spearman’s rho = 0.058, p = .40, and for PHQ8 Spearman’s rho=-0.058, p = .39).

### Adverse childhood experience score (ACE-Q)

Of the 228 youth who completed this instrument, 45% of youth had a score of zero, 26.3% had a score of 1, 13.6% had a score of 3, and 15% had a score ≥ 3, for a mean of 1.11. There was no correlation between cumulative dog exposure and ACE-Q score (Spearman’s Rho = -0.023, p = .7288). Both PHQ8 and SCARED41 scores were highly correlated with the ACE-Q score for the 228 youth who had ACE scores (between ACE-Q score and PHQ8, Spearman’s Rho = 0.284, p < .0001; and ACE-Q and SCARED41, Spearman’s Rho = 0.155, p = .019). There was no correlation between CABS and ACE-Q scores.

## Discussion

Exposure to pet dogs in childhood was negatively associated with the incidence of adolescent MH diagnoses, an association that was accentuated by high levels of attachment to the pet. Even at the crude measure of exposure (Y/N pet dog(s) at baseline), pet dogs were negatively associated with the risk of any MH diagnosis (p = .04). The most attached pet was significantly associated with a decreased risk for anxiety diagnosis (HR = 0.57, p = .006, 95% CI 0.38–0.85) and any MH diagnosis (HR = 0.64, p = .013, 95% CI 0.44–0.91). However, we found no significant relationship between any measure of pet exposure and more severe MH diagnoses for which psychotropic medications were prescribed.

During the eight year follow-up, 130 subjects lost 1 or more pets to death which can have a significant effect on mental health. However, this study was not structured to quantify this effect because the variability in time lapsed between pet death and when PHQ9 was administered precluded analysis of the impact of pet loss.

Despite the suggestion that neglected and abused children may be more likely to trust and attach to dogs [18], we found no correlation between cumulative dog or cat exposure and ACE-Q score, or with CABS and ACE-Q. This finding is consistent with a recent study showing that emotional closeness with a childhood pet dog did not moderate a significant association between ACE score and mental health [52].

Given the observational nature of the study, the possibility that pet dog exposure is simply a marker for a child and/or environment that is less vulnerable to mental illness must be considered. Understandably, a randomized trial of pet dog or cat ownership is neither feasible nor ethical. Given these two facts, the possibility of a causal link between pet dog exposure and reduced probability of MH diagnosis remains speculative, as the underlying mechanism for this relationship is unknown, although several have been proposed. Theoretical frameworks include HAI effects on dampening of the HPA axis in response to stress [53]. For example, dog owners interacting with their dogs have significant increases in plasma oxytocin levels that, in turn, decrease cortisol levels [54]. Another proposed mechanism is that pets transfer beneficial microbes to infants and young children that increase the diversity of their microbiome thereby improving the gut–microbiota–brain axis [55–57]. While this study was not designed to test underlying mechanisms, it provides further evidence of a positive relationship between pet dogs and youth mental health.

The COVID19 pandemic occurred 3/7/20 shortly after study start date 2/1/20, so it is important to consider how this context affected study results. Several studies have documented that the prevalence of anxiety, depression, and other MH problems among children and adolescents doubled during the pandemic [12, 58, 59]. This pandemic effect would presumably affect both subjects with and without pets equally in our study and therefore not bias the results. Next, there is some evidence that the pandemic initially reduced psychotropic medication prescriptions, but then later was associated with an increase in antidepressant prescriptions. These findings from a population-based study of 0 to 18 year olds in Manitoba, Canada, were attributed to lock downs in March 2020 that led to a decline in psychotropic medication use. This decline was followed by an increase in new-onset anxiety or depression during the pandemic that led to increased antidepressant prescription [60]. Again, these potential effects are likely equally distributed across our study population, and therefore would not bias our study outcome of MH diagnosis associated with a psychotropic prescription.

### Strengths

HAI research has been criticized for the lack of longitudinal studies, small sample sizes, poorly defined HAI instrument validity and reliability, and heterogeneous outcome assessment [24–28]. Our study addresses these weaknesses in the rigor of prior studies as follows. We leveraged the study results of our cross-sectional sample of children recruited 8 years prior to conduct a naturalistic, longitudinal analysis of the relationship between pet dog and cat exposure and the development of youth mental illness reflected by MH diagnosis. The study was strengthened by minimizing the biases associated with using only one outcome, e.g. parent or self-report of MH symptoms, by testing many potential covariates for confounding, and adjusting the final multivariate analysis for known confounders, i.e. income and baseline SCARED5 ≥ 3, that were associated with both pet ownership and MH diagnosis.

### Limitations

Our study was observational and nonrandomized. While we adjusted our analysis for demonstrated confounders, unmeasured confounders (e.g., pandemic-related variable school attendance, social isolation, etc.) may have influenced results. The stress of the pandemic may have limited parents’ willingness to participate in the follow-up interview and thereby contributed to non-response bias for data obtained by interview. Responders were more likely to have had a dog at baseline and to have an MH diagnosis during follow-up than were non-responders, however it is difficult to assess how these imbalances affect our study results. Measuring cumulative pet exposure may be subject to recall bias especially over long time intervals, thus calling into question the precision of duration of pet exposure reported by parents in this study. However, the exposure variable of pet dog exposure (Y/N) at baseline was not subject to recall bias, yet this model showed that pet dogs were negatively associated with the risk of any MH diagnosis (p = .04).

This study was conducted in a rural MHPSA, where limited access to MH services (exacerbated by the pandemic) implies a higher likelihood of lack of MH diagnosis or the potential for delayed or mis-diagnosis. Although MH diagnoses may be subject to mis-classification, under reporting, and are sometimes not reflective of full MH outcome assessment [37], they do signify significant impairment and in this study serve as a marker for MH disorder. These MH diagnosis limitations led us to use trans-diagnostic categories of *any MH diagnosis* that combines common MH disorders, but is less specific and does not inform what the underlying mechanism of the youth pet dog effect might be. Lastly, EMR data are collected in the context of providing healthcare and may be subject to within visit factors that influence documentation and accuracy of diagnosis and history of prior medications [61].

## Conclusion

Cumulative exposure to a highly attached pet dog or cat during childhood is associated with reduced risk of adolescent MH disorders. Although the underlying mechanism for this association is unknown, this study adds to the growing evidence supporting the potential benefits of companion animal interaction for youth socioemotional development. This study also underscores the need to measure and account for the level of child or youth pet attachment and duration of exposure, rather than simply studying the pet ownership [62].

## Data Availability

Materials may be requested by contacting the corresponding author (anne.gadomski@bassett.org). The database contains patient data and will not be shared.

## List of abbreviations

ACE-Q: Adverse Child Experiences Questionnaire.
ADHD: Attention Deficit Hyperactivity Disorder.
CABS: Companion Animal Bonding Scale.
EMR: electronic medical record.
HAI: human animal interaction.
HR: hazard ratio.
ICD-9: International Classification of Diseases, Ninth revision.
ICD-10: International Classification of Diseases, Tenth revision.
MH: mental health.
MHPSA: Mental Health Professional Shortage Area.
MSPSS: Multidimensional Scale of Perceived Social Support.
PHQ-8: Patient Health Questionnaire 8-item.
SCARED: Screen for Child Anxiety Related Emotional Disorders.
